# Neurosurgery As An Aptitude

**DOI:** 10.25122/jml-2019-1002

**Published:** 2019

**Authors:** Victor Lorin Purcărea

Graduate of the Institute of Medicine and Pharmacy in Bucharest in 1964, medical doctor, title obtained in 1974 with the doctoral thesis “Current therapeutic aspects in pituitary adenomas”, neurosurgeon since 1979, he was the Head of Neurosurgery I Department, “Bagdasar-Arseni” Clinical Emergency Hospital, coordinator of the Neuroscience Research Department of “Bagdasar-Arseni” Clinical Emergency Hospital and has been professor of Neurosurgery since 1997. He has held prestigious positions such as vice-president of the World Federation of Neurosurgical Societies (WFNS), corresponding member of the Brazilian Academy of Neurosurgery, corresponding member of the Romanian Academy of Sciences, vice-president of Academia Multidisciplinaria Neurotraumatologica (AMN), and corresponding member of Romanian Academy of Medical Sciences.

He has been 1^st^ Grade Researcher since May 2009.

His scientific activity includes many articles published in ISI (Clarivate) indexed journals such as Neurosurgical Review, Surgical Neurology, Neurosurgery, Acta Neurochirurgia, etc., as well as articles published in journals indexed in international databases (PubMed): Neurology Psychiatry Neurosurgery; Pediatrics; Neurology studies and research; Journal of Pediatrics, obstetrics and gynecology; etc. Moreover, he has also published over 100 articles in Romanian journals and is the main author of 35 treaties and monographs published in Romania and abroad, such as **Pediatric neurosurgery pathology**, Academia Publishing House, for which he received **“Gh. Marinescu” prize of the Romanian Academy**. He is co-author of **21 neurosurgical and neurological treaties and monographs**. He is member of many editorial boards of international journals such as Child’s Nervous System, Springer-Verlag, Germany; Surgical Neurology, USA; Acta Neurochirurgica, Springer-Verlag, Vienna; Asian Journal of Neurosurgery, Nagoya, Japan; Journal of Medicine and Life, Romania; etc.

**Figure 1:**
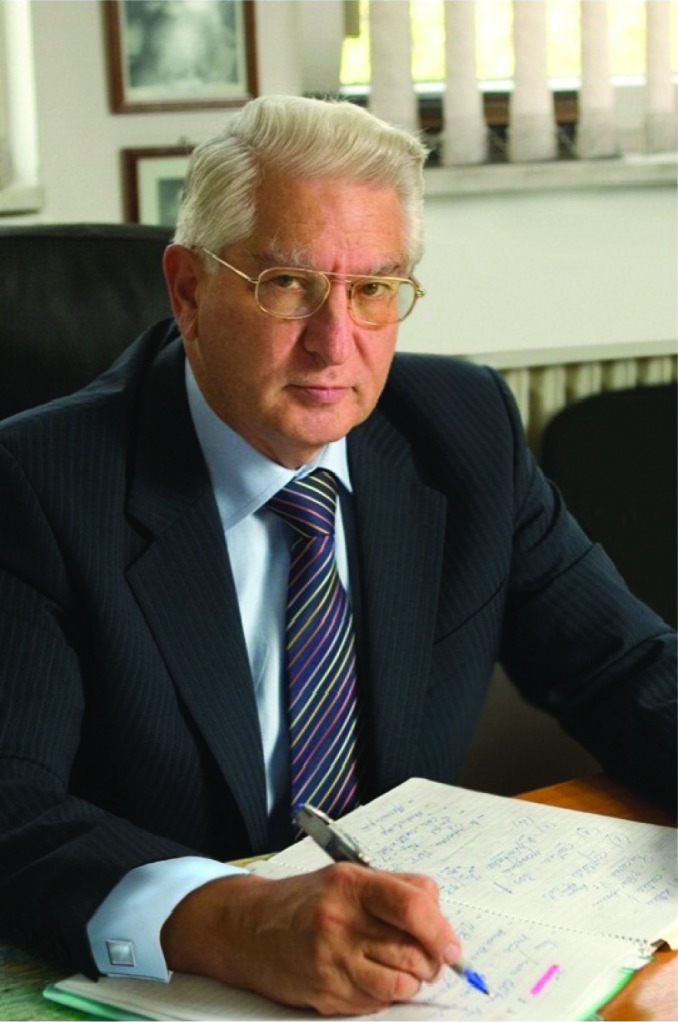
Prof. Alexandru Vlad Ciurea, MD, PhD

**Figure 2:**
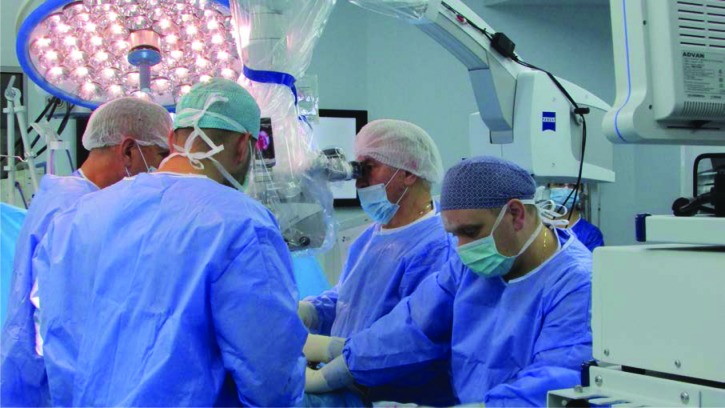
Operating room

**Figure 3:**
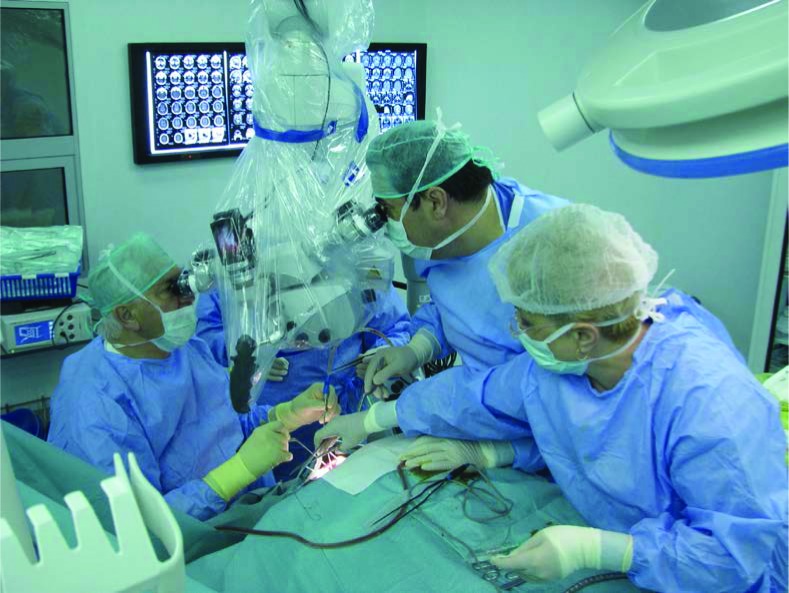
Prof. Alexandru Vlad Ciurea, MD, PhD (left) during a surgery

**Figure 4:**
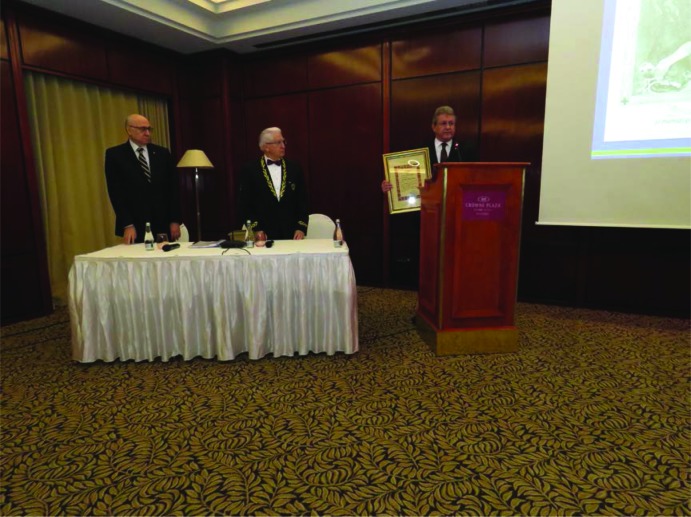
Prof. Alexandru Vlad Ciurea, MD, PhD at the ceremony during which he was awarded the honorable title “Commander of Order of Romania”

He founded the neuroscience research department of “Bagdasar-Arseni” Clinical Hospital in Bucharest and has led and been involved in many research projects.

He has collaborated with prestigious universities abroad such as the University of Virginia, USA, Karolinska University, Sweden, Marburg University, Germany, Messina University, Italy.

He holds a patent for “Unitub Drainage”, which he obtained in 2005. He applied the technique of ventriculo-peritoneal shunt-unitub drainage for the first time in Romania in 1985, currently being a procedure used as surgical treatment in children with primary and secondary hydrocephalus.

For his activity and remarkable results, he has been awarded many prizes such as the National Order “Faithful Service” as Commander. He was also awarded the Doctor Honoris Causa title by universities in Galaţi, Oradea, Chişinău, Iaşi, Piteşti, Constanţa and was Visiting Professor at 11 universities, such as Harvard University in Boston, INI in Hannover, Mercer University in Atlanta, etc.

In one of his latest articles, Prof. Alexandru Vlad Ciurea, MD, PhD, affirmed: “Even for the most gifted neurosurgeons and despite modern medical devices, or for psychologists and famous neurologists, the brain remains a command center that has its enigmas”. In an impressive ceremony that took place in Bucharest, on January 31, 2019, with a select audience, the Eminent Professor was awarded the honorable title “Commander of Order of Romania”. This order is rarely awarded only to prominent personalities with a major activity in promoting our country.

**Figure 5:**
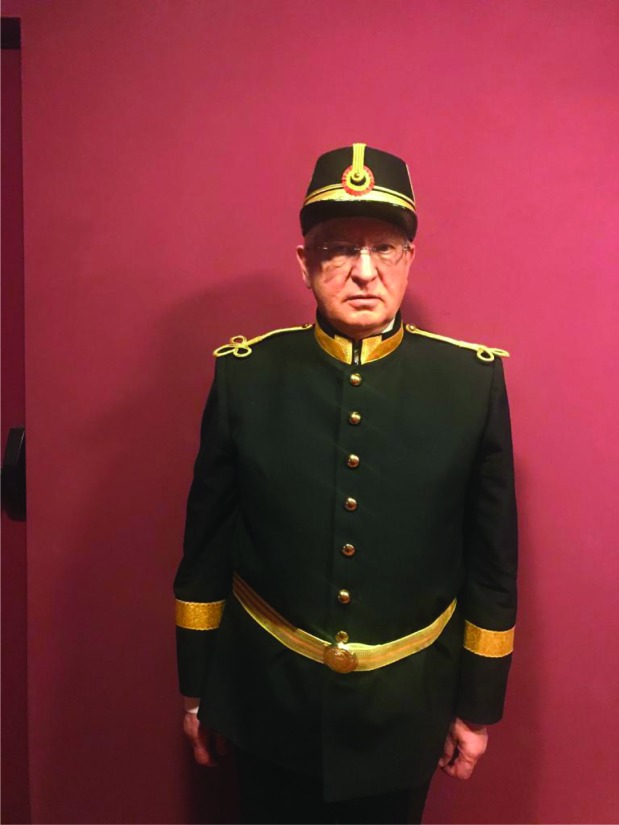
Prof. Alexandru Vlad Ciurea, MD, PhD at the entrance in “Atelier” Hall of the National Theatre in Bucharest

“As graduate of “Andrei Şaguna” National College in Braşov, I had the privilege of studying by following “the footsteps” of real patriots, people who participated in the great event of the Union! I was a student of the famous high school and I walked through the same corridors, passed through the same classrooms, through which many of the Union’s personalities stepped in”, justified the distinguished professor on March, 10 2019, at the entrance in “Atelier” Hall of the National Theatre in Bucharest, his wearing the high school uniform to what was going to be another new and fascinating lesson on health, titled “Alzheimer understood by everyone“.

The famous neurosurgeon stated, “Alzheimer is considered incurable, with a long pre-clinical period and a progressive course of the affection, from minor to severe forms of the disease. Many political, artistic and sports personalities have died from this disorder, and the main therapeutic method is prevention (diet, lifestyle, connections, socialization, music, tourism and positive thinking, etc.)”.

Undoubtedly, the overwhelming personality, the extraordinary power of work and especially his tenacity and will, doubled by a permanent opening to novelty and a continuous availability, have made Prof. Alexandru Vlad Ciurea, MD, PhD, a complete neurosurgeon.

Thank you for being, Prof. Alexandru Vlad Ciurea, MD, PhD!

